# U.S. Agro-Climate in 20^th^ Century: Growing Degree Days, First and Last Frost, Growing Season Length, and Impacts on Crop Yields

**DOI:** 10.1038/s41598-018-25212-2

**Published:** 2018-05-03

**Authors:** Meetpal S. Kukal, Suat Irmak

**Affiliations:** University of Nebraska-Lincoln, Lincoln, NE 68583 USA

## Abstract

Significant air temperature changes have occurred globally during the 20^th^ century, which are spatially variable to a considerable degree and these changes can have substantial implications in agroecosystem productivity. The agroclimate indicators that are responsible in these contexts are first fall frost (FFF), last spring frost (LSF), climatological growing season (CGS) length, and heat accumulation (growing degree days, GDD). We explore spatial and temporal trends associated with these indices across the continental U.S. (CONUS) during 1900–2014 using datasets collected at 1218 sites. On average, FFF has been occurring later (by 5.4 days century^−1^), and LSF has been occurring earlier (by 6.9 days century^−1^), resulting in the average lengthening of the CGS (by 12.7 days century^−1^). Annual GDD has been increasing by 50 °C century^−1^. We also report trends for agricultural belts and climate regions. We developed relationships between county-level crop yields vs. agroclimate changes and found that all crops (maize, soybean, sorghum, spring wheat, winter wheat, and cotton) responded positively to a lengthened CGS, while responding negatively to increase in GDD, except cotton. Overall, we find that the observed changes in agroclimate, were beneficial for crop yields in the CONUS, albeit some crop and region specific exceptions.

## Introduction

Natural and anthropogenic variability and trends in climate during the 20^th^ century have been associated with increases in air temperatures at the earth’s surface, with a recorded global terrestrial warming of 0.74 °C^1^. These trends are highly variable in terms of geography, and regions have to be individually evaluated for determination of the site-specific impacts on environment, agriculture, public health and a range of areas that are prone to temperature changes. However, addressal of these impacts, especially in the area of agricultural production, cannot merely rely on trends in average temperatures. Crop physiology during the growing season is primarily driven by accumulation of heat units, rather than average air temperatures. Heat accumulation is responsible for, and hence, is extensively employed to predict crop growth and development, yield potential, crop water uptake and stress^2^. The most common temperature-based index used for these activities is usually referred to as growing degree days (GDD) or thermal units, or thermal time. Another crucial factor that governs the agricultural food production in terms of the sensitivity of plants to frost is the timing of frost events and the frost free-period or the climatological growing season. Heat accumulation and frost characteristics, hence are the two derived aspects/proxies of air temperature and detailed information of spatial and temporal nature of these indices are required when climate change vs. agricultural food production issues are concerned.

A detailed analysis on the dynamics of these two agroclimate indices would aid in answering questions such as: (i) is the warming climate also leading to greater heat accumulation in shorter periods, which would impact the phenological development of crops? (ii) Is the warmer climate delaying or advancing frost days, which would result in alteration of the frost-free period for the actual crop growing season? (iii) Do we need to adapt or shift towards longer or shorter maturity crops as one of the mitigation strategies? (iv) Do we need to plan for more-frequent irrigation events given the higher crop water demand owing to the warming? (v) Do we need to adjust and adapt in order to take advantage of beneficial agricultural environmental conditions (or regions) and mitigate the detrimental conditions to sustain agricultural production? (vi) Do we need to shift crop planting dates to allow for appropriate crop maturity and consequently, maximum yields? An effort aimed at developing a strong knowledge base of observed historical trends is indispensable to be able to answer these questions with scientific analyses and evidence.

Some studies have investigated the changes in these agro-climatological indices in the recent past for the United States^3–5^. The results from these studies have been consistent in reporting a general lengthening of the growing season or the frost-free period, by about 2 weeks during the 20^th^ century in the continental U.S. (CONUS), by about a week for North America during 1950–2011, and by 0.89 days decade^−1^ during 1901–2009 for the Northern Hemisphere. The studies concur that this lengthening has occurred faster during the latter half of the century. Growing degree days and its long-term variability have not been discussed, at least, as widely as frost dates and frost-free periods at the national (USA) scale. In one study^4^, it was reported that thermal time (or GDD) has increased in the western U.S. and decreased in the eastern U.S. from 1951–2000. Our study attempts to bridge the gap between two interdependent characteristics of crop growing conditions (GDD and frost-free period), by addressing them under a common framework.

The majority of the previous efforts in this direction have two major limitations. Firstly, most of these look at agroclimate trends starting mid-century (around 1950), which is justified due to non-availability of digitized climate data in the past. However, as of now, century-long datasets of air temperatures are available, although not for as many locations as post-1950. This provides us with unprecedented opportunities to investigate long-term (>100 years) trends in agro-climate. Secondly, the aforementioned studies do not address the potential implications of these resulting trends for various cropping systems and regions, especially in a quantitative manner, given that the trends are highly spatially dynamic in nature and varying susceptibility of different crops to these changes.

This study is unique in the aspects that: a) It spans a period of 115-years (1900–2014), which is a decade longer than the longest period of study in the literature; b) It reports the trends in agro-climate with respect to six national agricultural belts and quantitatively explore how these trends could affect agricultural yields in these regions. Specifically, this study presents research efforts to investigate the changes in growing degree days throughout the year and how these changes vary spatially and temporally across the conterminous United States (CONUS) during this period, which can significantly contribute to scientific literature and enhance our understandings of these dynamics and can contribute to developing effective local, regional, and national agriculture vs. climate interactions strategies and policies to enhance national agricultural productivity. Also, the first fall frost (FFF) and last spring frost (LSF) and finally, climatological growing season (CGS) were evaluated for trends, over the same spatial and temporal scales. The study also presents these historical changes in these agro-climatological indices for major agricultural belts in the CONUS namely maize (*Zea mays*), soybean (*Glycine max*), cotton (*Gossypium*), winter wheat (*Triticum aestivum*), and spring wheat (*Triticum spelta*) belts to serve as monitoring references as defined by NOAA. The study reports all the major and quantitative results as information depicted on maps for better interpretation. Finally, it also presents preliminary relationships among the resulting trends in agro-climate and major crop yields in their respective growing regions, which can provide invaluable data and information to support policy decisions.

## Results

### Growing degree days/heat accumulation

**Figure 1:**
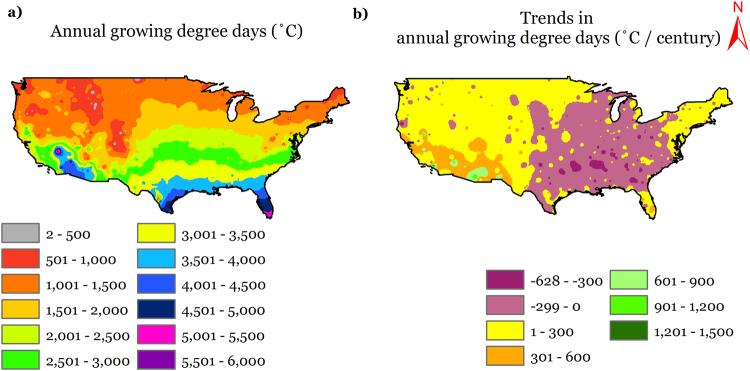
Map showing (**a**) spatial distribution of long-term average annual accumulated growing degree days (AGDD); (**b**) temporal trends in annual accumulated growing degree days during the period 1900–2014 across CONUS. We created the maps using ESRI ArcMap 10.4.1 software http://desktop.arcgis.com/en/arcmap/.

**Figure 2:**
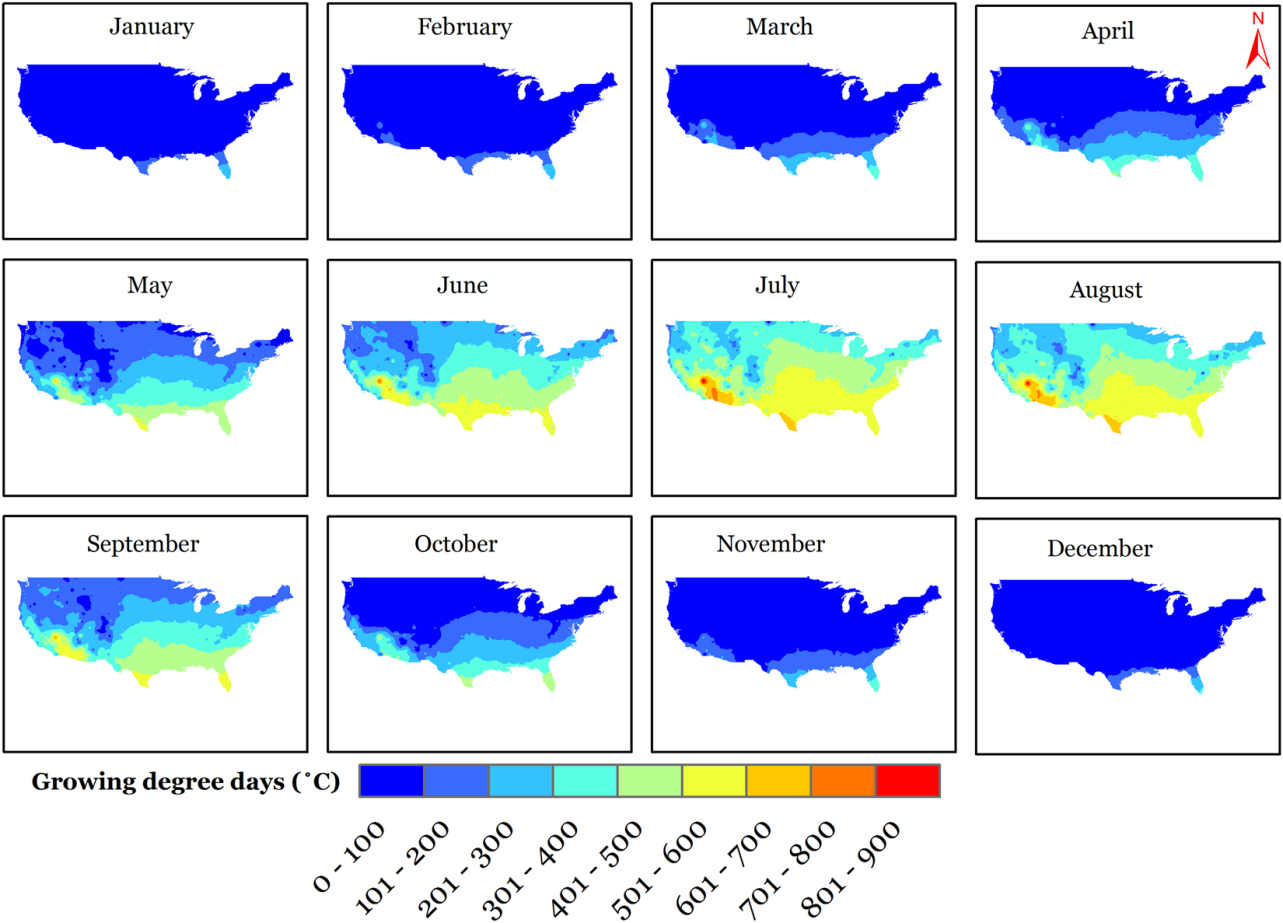
Spatial distribution of long-term average accumulated growing degree days (AGDD) for different months of the year. We created the maps using ESRI ArcMap 10.4.1 software http://desktop.arcgis.com/en/arcmap/.

Growing degree days (GDD) accumulated for the annual period averaged for the period 1900–2014 is presented in Fig. 1a. The definition of GDD we employed does not impose an upper limit to daily GDD, and hence would include the contribution of extreme heat days into the accumulated GDD magnitudes. Generally, the agricultural GDD (AGDD) follows a clear latitudinal pattern with increasing magnitudes as we move from north to south with some exceptions such as Rocky Mountains ranges, which are lower in AGDD magnitudes than their surroundings and Mojave and Sonoran deserts in the west, which are naturally higher in AGDD magnitudes than their surroundings due to high air temperatures. The maximum and minimum station-observed average AGDD were observed at Key West International Airport, Florida (5722 °C) and Dillon, Colorado (249 °C), respectively. Figure 2 presents the long-term average AGDD for different months of the year. On a national average basis, monthly GDD progresses from a minimum of 15 °C in January to a maximum of 423 °C in July and decreases thereafter. Site-specific values vary largely from the national average; nevertheless, they follow similar monthly trends.

**Figure 3:**
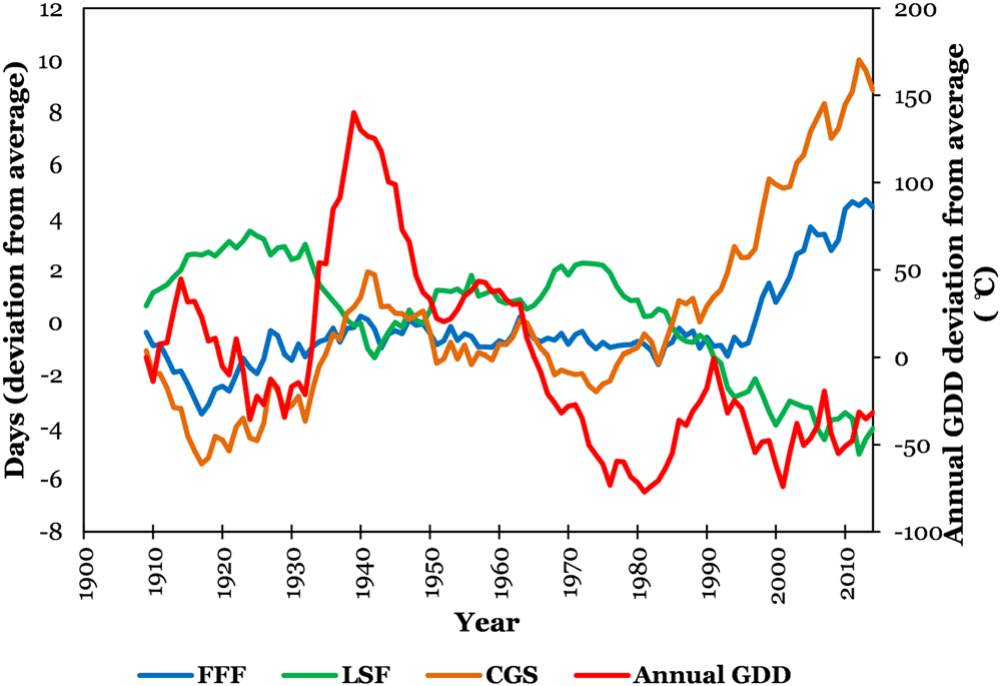
National-level trends in first fall frost (FFF), last spring frost (LSF), climatological growing season (CGS) and annual accumulated growing degree days (AGDD). The trends are shown using deviation of each series from average from 1900–2014. Each series represents a 10-year running average.

**Figure 4:**
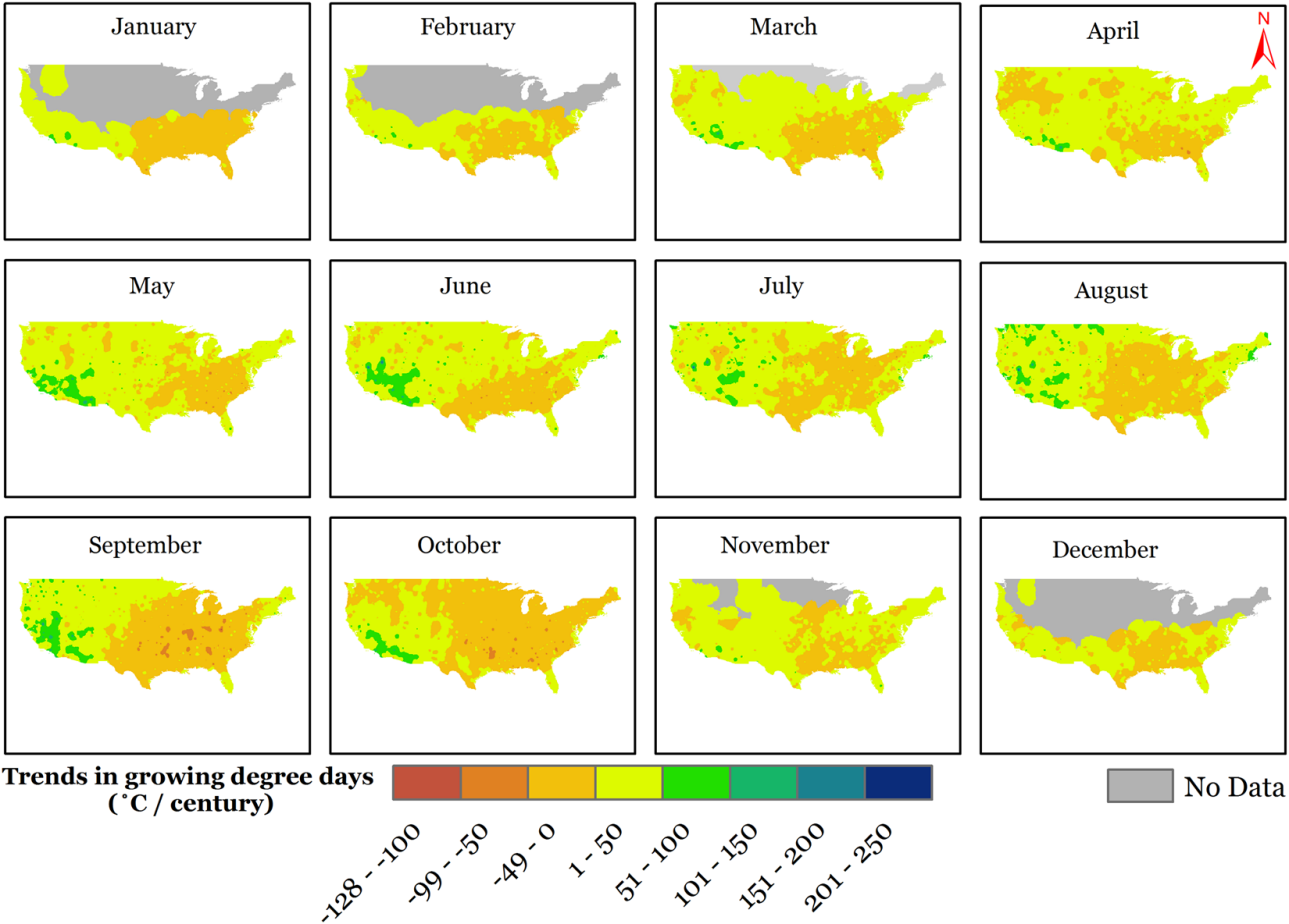
Map showing temporal trends in accumulated growing degree days (AGDD) for different months of the year during the period 1900–2014. We created the maps using ESRI ArcMap 10.4.1 software http://desktop.arcgis.com/en/arcmap/.

The annual AGDD trends (deviation from mean annual AGDD) in time domain (1900–2014) are presented on a national scale in Fig. 3. The deviation was initially close to zero, which rose to a positive maximum in 1939 and thereby started declining into negative deviations until the end of the study period in 2014. The national time series is derived from observed data at 1218 sites, and presents a national changes in AGDD, but it should be acknowledged that the constituent sites show highly variable trends and relying on a national series can conceal regional variations. These regional differences in annual AGDD trends can be seen in Fig. 1b. Overall, the AGDD trends can be divided into two regimes; positive trends in western U.S., and negative trends in eastern U.S., with the major exception of positive trends in the northeastern U.S. Southwestern U.S. generally shows relatively higher positive trends than those in the west. There are several regions throughout the nation which show trends in opposite direction than the surroundings (pink spots in yellow region and vice-versa in Fig. 1b), which imply that the geographic patterns in temporal trends are highly variable. The site-specific extremes in temporal trends were 1276 °C century^−1^ (in California) and −632 °C century^−1^ (in Mississippi), while the national average temporal trend was 19 °C century^−1^ because of the countering positive and negative changes in AGDD. These trends in annual AGDD result from varying trends in AGDD during different months and could be distributed in a particular manner during the year. To answer this, we present Fig. 4 that enables us to present and interpret the temporal trends in AGDD on a monthly time scale. When averaged nationally, positive (increasing) monthly AGDD trends were observed for all months, except for January, September, and October, which had negative (decreasing) trends. These trend values, however, cannot be compared amongst various months fairly because the AGDD climatology is considerably different for each month. In other words, trends in summer would be higher due to more accumulation of GDD’s from higher air temperatures and would be lower in winters for the opposite reason, and hence it would not be valid to make assessments by comparing these trends. To resolve this, we normalized the observed trend for each month by the average AGDD during that month, which enables us to make comparisons among trends during different times of the year. The resulting maximum increasing trends were found in February, followed by November and December, whereas the decreasing trends were the highest in January (2.5 times greater in magnitude than the maximum increasing trend) followed by October, while the rest of the months show comparable trend magnitudes (Figure S1). Although this analysis provides valuable insights into national-level monthly AGDD trends, it still does not provide information about regional-level dynamics in monthly AGDD trends. Figure 4 shows that significant spatial variability in monthly AGDD trends exists within the nation. In scenarios such as these, national-level trends tend to mask the finer-scale variability observed in Fig. 4. In other words, an indicator may show considerable spatial variability when studied at finer scales, but this variability can get concealed, when the same indictor is studied on a coarser national scale. Thus, relying on national-level interpretations can potentially be misdirecting. Instead, we recommend referring to the developed maps to consult for site-specific trends, rather than relying on national scale information for assessments.

### Annual frost dates

**Figure 5:**
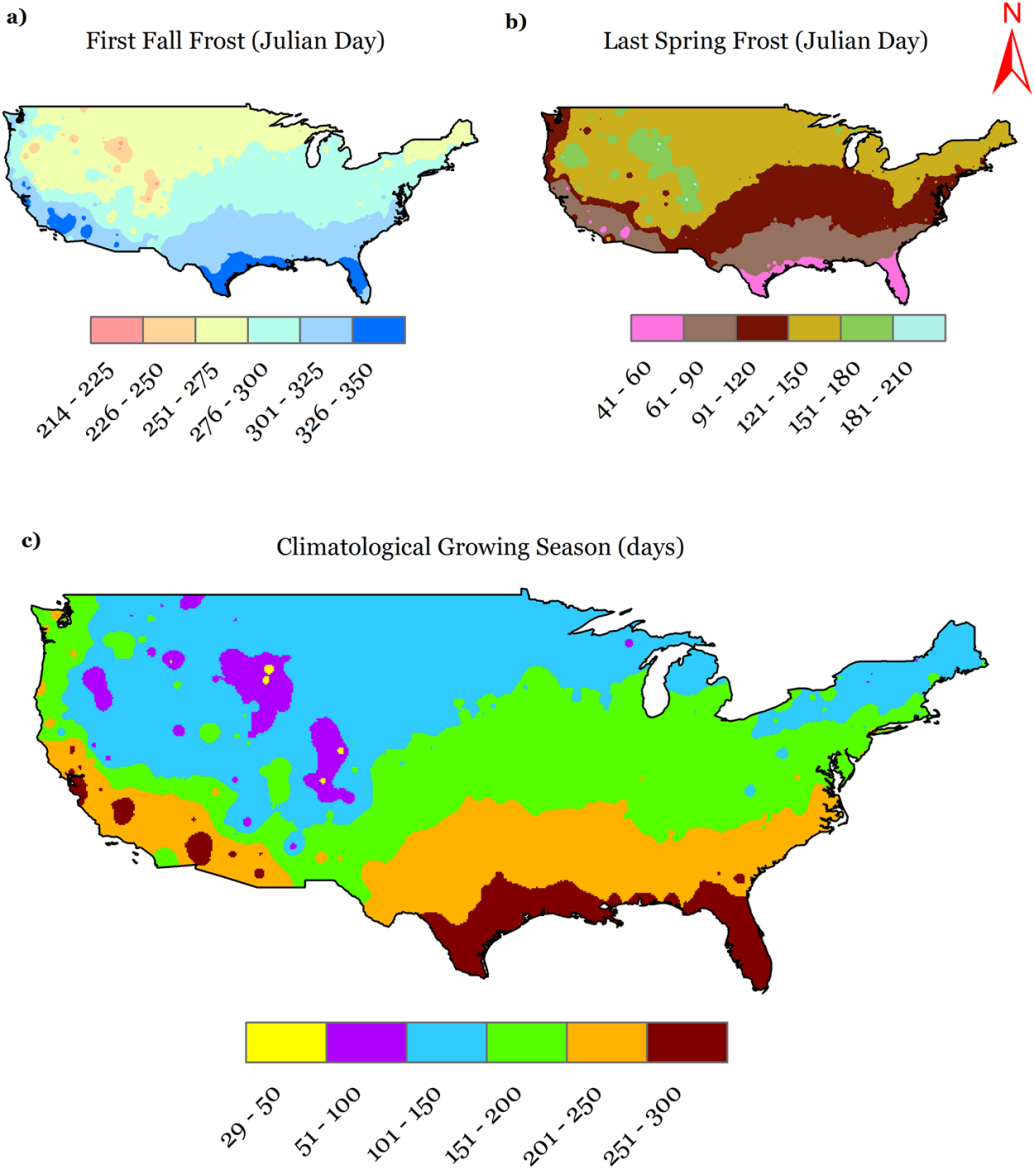
Spatial distribution of long-term average (1900–2014) (**a**) first fall frost (FFF); (**b**) last spring frost (LSF) and (**c**) climatological growing season (CGS) across CONUS. We created the maps using ESRI ArcMap 10.4.1 software http://desktop.arcgis.com/en/arcmap/.

The occurrence of first fall frost (FFF) and last spring frost (LSF) in terms of day of the year for the CONUS is presented in Figs 5a,b, respectively. Both FFF and LSF follow a north to south latitudinal spatial trend, except some extremes in the Rocky Mountain ranges. FFF occurs later in the year as we move north to south, while LSF occurs earlier in the year as we move north to south. The national averages of occurrence of FFF and LSF were day of year (DOY) 286 and DOY 115, respectively. The magnitudes of FFF vary by about 125 days throughout the CONUS with extremes in Wyoming (DOY 214) and California (DOY 339). Similarly, occurrence of LSF varies by about 146 days in the nation with extremes in Colorado (DOY 187) and Florida (DOY 41).

**Figure 6:**
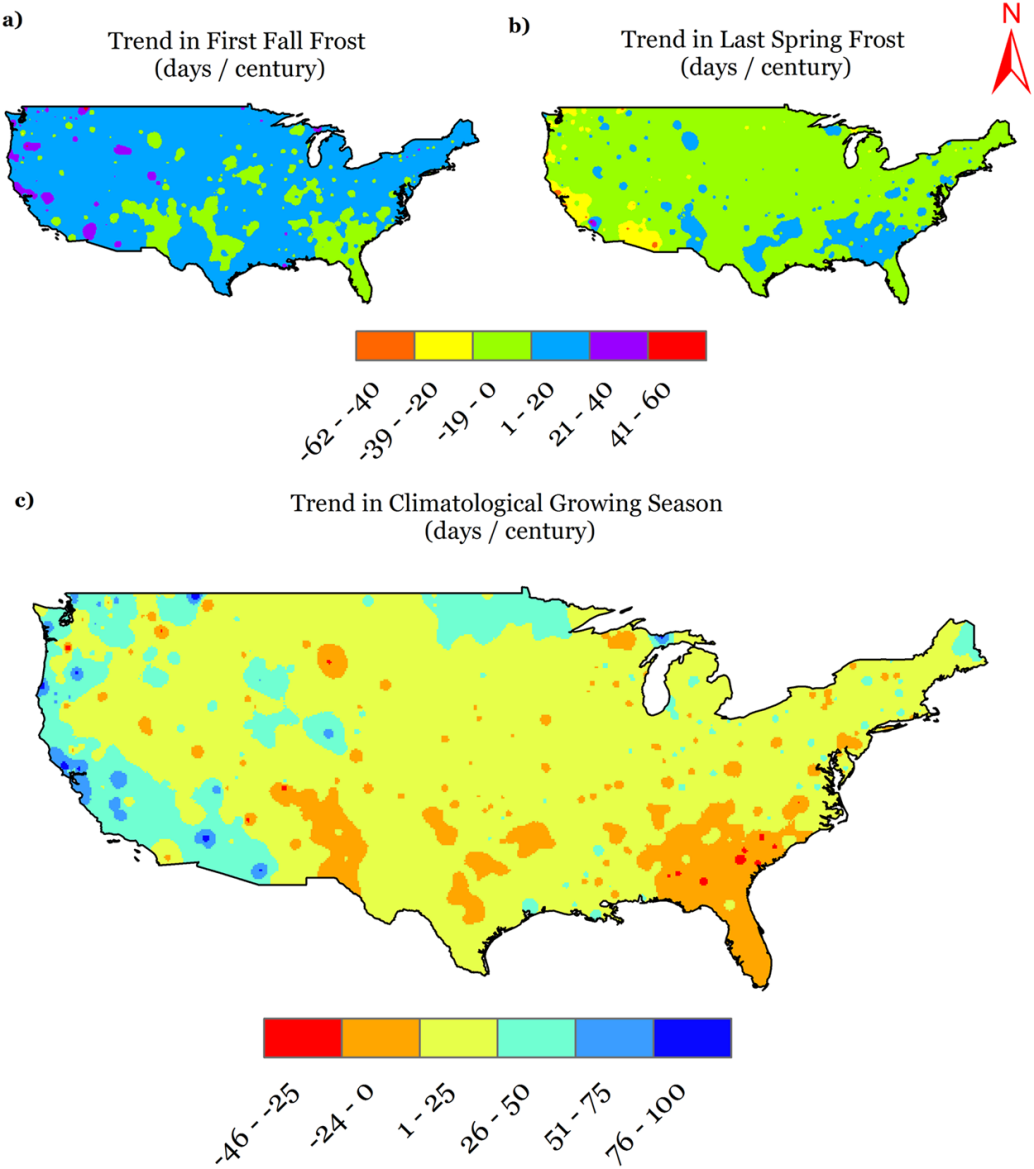
Temporal trends in (**a**) first fall frost (FFF); (**b**) last spring frost (LSF), and (**c**) climatological growing season (CGS) across CONUS during 1900–2014. We created the maps using ESRI ArcMap 10.4.1 software http://desktop.arcgis.com/en/arcmap/.

The national level deviations in the occurrence of the FFF and LSF are shown in Fig. 3. The dates followed trends that are variable in time; with two periods with considerable deviations from the national average. Between these two periods, both dates occurred closer to the average, especially FFF (LSF occurred mostly later than average). One of these periods was before 1930, when the fall frosts occurred earlier (2–3 days) and the spring frosts occurred later (3–4 days). The other period was post-1990 for FFF and post-1970 for LSF, where FFF started occurring later and LSF started occurring earlier. On a national basis, the temporal trends in FFF and LSF were about 5 days century^−1^ (later occurrence) and −7 days century^−1^ (earlier occurrence), respectively. To be able to segregate and decipher national trends, we computed these trends throughout the CONUS at a spatial scale and the results are presented in Figs 6a,b. For FFF, the majority of the nation has positive trends that scale up to 20 days century^−1^, which implies that FFF has occurred up to 20 days later over the century, while even greater trends exist (up to 40 days century^−1^) in small parts of the western U.S. In contrast, there are some regions, for example in the Midwest, southeast, south and central southwest (colored in green), which show negative trends (up to −19 days century^−1^), which implies that FFF has occurred earlier in these regions by a magnitude of up to 19 days over a century. On the other hand, LSF occurrence is shown to be earlier for the most part of the nation (by up to −19 days century^−1^), along with some parts in southwestern U.S., which show even greater rates of negative trends (up to −39 days century^−1^). Delays in LSF (by up to 20 days century^−1^) were observed in some scattered parts in the west, southwest, south, and southeast. The maps developed in this section can be an invaluable resource to the scientific community as well as for decision and policymakers and resource managers and can be employed to generate quantitative information on FFF and LSF trends at any site in the CONUS.

### Climatological growing season

Climatological growing season, by its definition, is the difference between the LSF and FFF and is reported in Julian days, same as LSF and FFF. Hence, any spatial and temporal changes in one or both of the annual frost dates will trigger a change in CGS as well. Figure 5c presents the spatial patterns associated with CGS across the CONUS, and we find that it follows a north to south (increasing) trend, which is similar to AGDD, FFF, and LSF in its latitudinal pattern. The national average CGS is about 170 days, and it extends from a minimum of 29 days in Wyoming to a maximum of 289 days in California.

The national time series for deviation of CGS from national average (Fig. 3) exhibits a sharp increase from about a 5-day shorter CGS around 1915 to a 2 day longer CGS around 1940, a gradual decrease thereafter until a 3-day shorter CGS around 1975, and finally, a sharp increase until a 10-day longer CGS in 2014. Overall, considering the temporal changes in CGS on a spatial level (Fig. 6c), the majority of the CONUS experienced trends towards lengthening of the climatological growing season by about 25 days century^−1^, while there were regions such as along the west coast and northern plains that showed even greater lengthening trends up to 75 days century^−1^. However, some regions in the southeast, south, and central southwest show trends towards shortening of the CGS by about 24 days century^−1^. The extremes in observed station trends in CGS were in Montana (a lengthening of 96 days century^−1^) and Washington (shortening of 47 days century^−1^), while on a national scale, lengthening of the CGS was observed at the rate of about 12 days century^−1^.

### Trends based on agricultural belts and geographical zones

**Figure 7:**
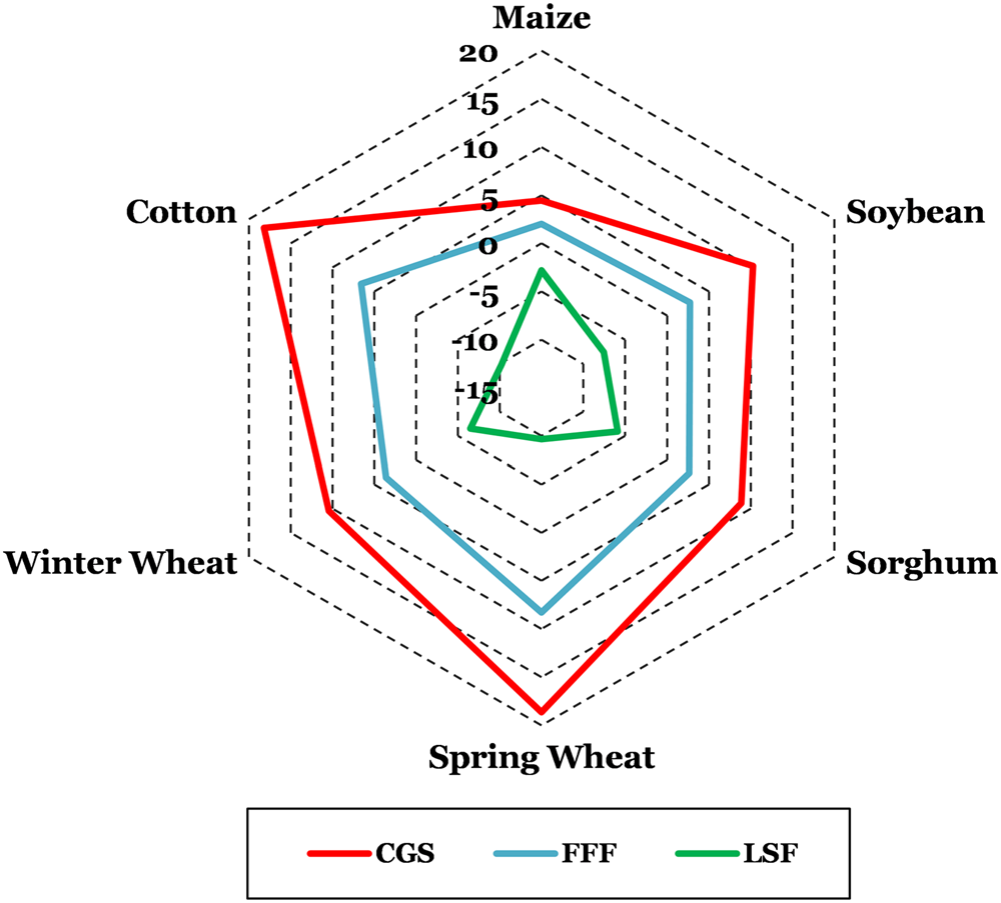
Trends in first fall frost (FFF), last spring frost (LSF), and climatological growing season (CGS) during 1900–2014 representative of each U.S. agricultural belt.

Crop-specific and climate region-level statistics were extracted from spatial information on the trends in FFF, LSF, and annual and monthly AGDD and are presented in Tables 1 and 2, respectively. Based on the statistics, we found that for all of the agricultural belts, there was an observed lengthening of the CGS, and the highest of these trends were for spring wheat and cotton belts, while maize belt experienced the least rate of CGS lengthening. These increases were because of delays in FFF and earlier occurrence of LSF, but the more dominant of the two shifts was the one in LSF, which shifted at a rate higher than FFF for all of the agricultural belts (Fig. 7). Although, crops such as winter wheat and spring wheat have different growing seasons than the rest of the summer crops, frost free days are still very important attributes of the growing periods of all these crops for growth and development and for their physiological functions for producing grains. Annual AGDD, was observed to have positive trends for spring wheat and cotton belts, but negative for maize, soybean, and winter wheat. Heat accumulation during the crop growing season (between planting and harvesting dates of each crop, which are presented in the Supplementary Table S1) decreased for all crops, except for spring wheat. This decrease in crop GDD was highest for winter wheat, followed by sorghum. Further, if we consider the monthly AGDD trends for the crop-specific growing season, it is interesting to note that both maize and soybean have trends such that during the early part of the crop growing season (April, May, June), AGDD shows increasing trends, while decreasing trends are observed for the rest of the season until near harvest in October. Also, these negative trends are greater for the soybean belt than maize belt. However, for cotton and spring wheat, the AGDD trends were positive for the entire growing season. Lastly, winter wheat belt was shown to have variable trends during its growing season, with dominantly negative trends for the initial part, from September until March, and positive trends thereafter until near-physiological maturity in July–August.

**Table 1:** Trends in agroclimate indices for the major CONUS agricultural belts.

Variable	Units	CONUS Agricultural Belts					
		Maize	Soybean	Sorghum	Spring Wheat	Winter Wheat	Cotton
CGS	Days century^-1^	4.5	10.3	8.9	18.7	10.5	18.2
FFF		2.0	2.8	2.7	8.3	3.6	6.6
LSF		−2.8	−7.6	−5.9	−9.7	−6.5	−10.2
Crop Growing season AGDD	Degree C century^−1^	−79.0	−57.1	−123.5	106.0	−187.6	−38.4
January AGDD		N/A	−2.6	N/A	0.0	−0.7	−3.7
February AGDD		0.0	−0.8	N/A	0.0	−0.1	5.3
March AGDD		3.1	−2.2	−0.2	0.5	−0.2	8.4
April AGDD		7.3	3.4	3.2	1.9	3.1	9.8
May AGDD		2.9	3.6	10.0	13.1	7.6	18.7
June AGDD		11.5	10.7	3.6	19.4	6.6	14.2
July AGDD		−0.3	−6.6	−2.2	18.1	2.0	9.8
August AGDD		−2.0	−12.2	−9.1	22.2	2.0	14.7
September AGDD		−15.8	−19.8	−19.8	18.0	−8.4	13.9
October AGDD		−18.3	−21.5	−15.0	−3.4	−14.4	13.7
November AGDD		1.8	0.8	3.7	0.2	1.4	7.1
December AGDD		0.1	−0.6	N/A	0.0	0.2	0.2

Cells with N/A indicate that a trend could not be computed due to zero heat accumulation in those months.

**Table 2:** Trends in agroclimate indices for the CONUS climate regions.

Variable	Units	CONUS	CONUS Climate Regions								
			West	Upper Midwest	Southwest	Southeast	South	Ohio Valley	Northwest	N. Rockies and Plains	Northeast
CGS	Days century^−1^	12.7	27.0	14.5	13.7	−3.4	7.4	9.1	22.5	14.2	13.4
FFF		5.4	10.7	5.0	5.5	−0.7	3.0	2.9	11.2	6.2	7.6
LSF		−6.9	−12.4	−9.0	−8.3	0.6	−4.1	−6.5	−9.8	−7.4	−6.4
Annual AGDD	Degree C century^−1^	50.2	236.4	−21.6	279.2	−78.8	−84.8	−129.1	111.3	77.8	86.2
January AGDD		−1.0	10.5	N/A	4.3	−12.3	−7.6	−0.7	0.2	N/A	N/A
February AGDD		1.2	11.4	N/A	5.6	−2.0	−1.9	−0.1	0.1	N/A	N/A
March AGDD		1.7	20.3	0.4	13.6	−9.8	−7.0	−1.6	0.8	0.7	1.0
April AGDD		4.5	14.3	3.8	17.1	−6.3	−1.6	5.5	−1.7	2.5	10.1
May AGDD		10.9	36.0	9.0	31.6	−15.1	7.4	−6.5	9.6	10.5	12.3
June AGDD		12.5	40.5	15.4	34.2	−9.1	−5.2	−1.7	10.9	15.4	19.4
July AGDD		10.1	26.8	2.6	32.1	3.6	−3.4	−13.3	18.2	14.4	12.4
August AGDD		11.8	35.2	5.0	26.9	−1.2	−10.0	−13.4	30.0	20.4	26.4
September AGDD		2.6	47.9	−9.1	22.0	−20.3	−23.7	−34.6	33.2	15.6	1.0
October AGDD		−5.4	30.4	−12.6	17.1	−20.3	−17.7	−27.1	−0.7	−6.3	−8.2
November AGDD		2.9	8.7	N/A	8.0	2.9	2.1	1.2	0.7	0.2	2.8
December AGDD		0.5	3.8	N/A	2.3	1.3	−1.3	−0.3	0.1	N/A	0.2

Cells with N/A indicate that a trend could not be computed due to zero heat accumulation in those months.

From the climate region-based analyses, it was revealed that except the southeast region, all regions showed positive trends in CGS, positive trends in FFF, and negative trends in LSF. The highest rate of CGS lengthening and LSF advances were observed in the west, while the highest rates of FFF delays were in the northwest. Positive trends in annual AGDD were found in the west, southwest, northwest, northern Rockies and Plains, and northeast, while negative trends were found in the Upper Midwest, southeast, south, and Ohio Valley. Detailed statistics on monthly AGDD temporal trends are listed in Table 2. To present an overview of the agroclimate climatology, Supplementary Table S2 serves to provide long-term mean magnitudes of agroclimate indices for various U.S. climate regions. Using this information, the trends that have occurred in agroclimate indices can be related to the long-term mean spatial patterns.

### Crop yield-agroclimate relationships

**Figure 8:**
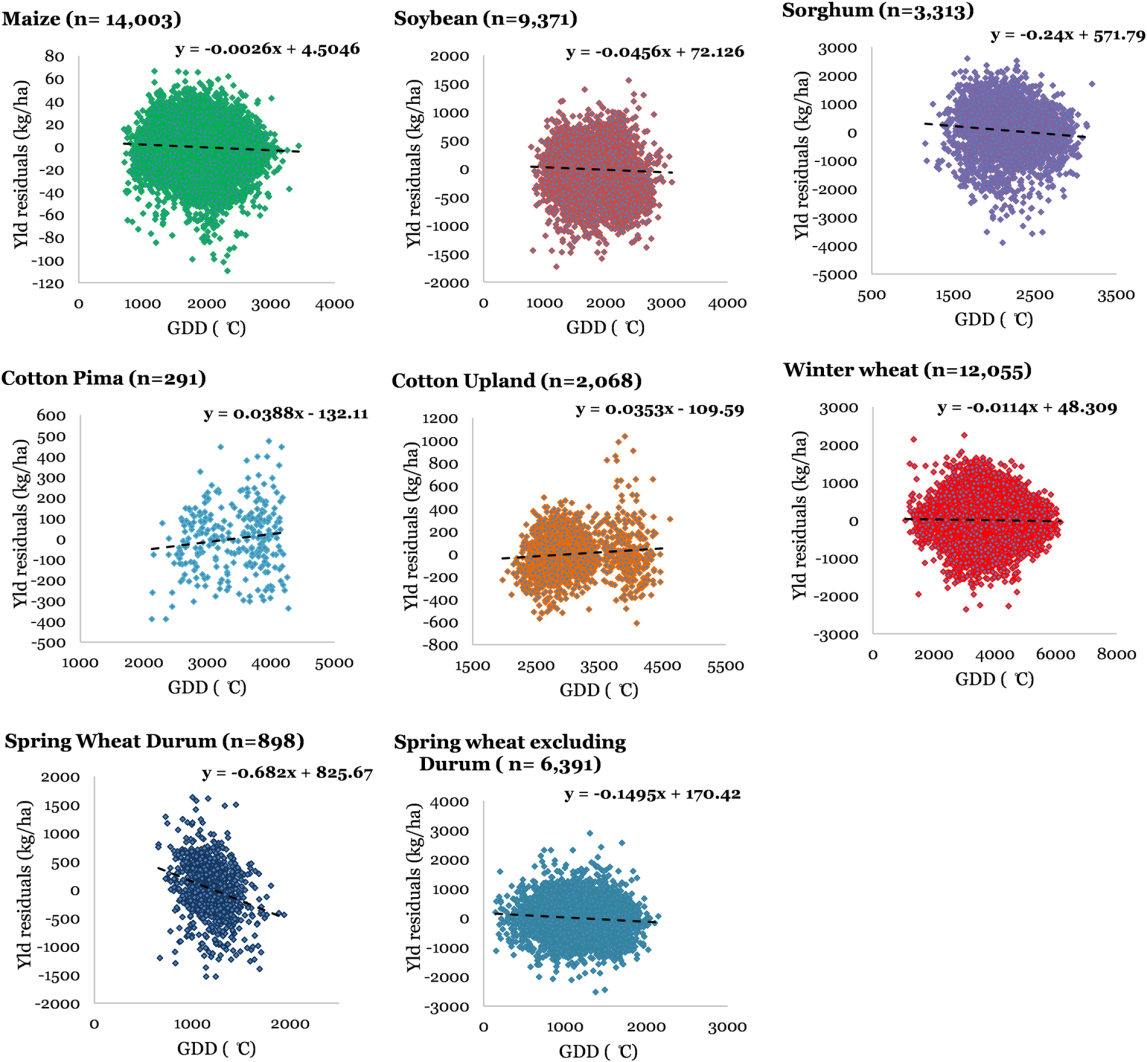
County-level relationships among yield and growing degree days (GDD) pooled nationally for maize, soybean, sorghum, cotton (pima and upland), winter wheat, and spring wheat (durum and non-durum). Each regression curve includes *n* number of site-years in the CONUS during 1900–2014.

We attempted to explore relationships between inter-annual variability associated with crop yields and GDD accumulated during a particular crop’s growing season on a county scale for the CONUS. We used crop yield residuals (against time) regressed against seasonal GDD to characterize these relationships. Figure 8 presents these functions for each crop for different number of site-years data. The slopes of these relationships were negative for all crops, except cotton (both Pima and Upland varieties), which showed positive slopes. Hence, we found that maize, soybean, sorghum, and wheat (spring and winter) yields, on a pooled national scale, demonstrated reduced yields in higher GDD site-years, whereas cotton yields show increased yields in higher GDD site-years. However, it has to be recognized that these national-level relationships can mask finer scale relationships due to data aggregation and normalization of any potential location-specific trends. These relationships include geographic (sites) and temporal (years) information aggregated into a single linear function and hence pools significant variability and potentially causes loss of information, which is demonstrated by high scatter and low coefficient of determination (R^2^) (<0.01 for all crops, hence not shown on the curves). It is also likely that the nature of these relationships varies among counties in the same region as well as between the regions, as a given crop can have different sensitivities and response to increasing GDD in different regions due to numerous factors, including geographic differences; soil type; crop varieties grown; climate; soil, crop, and water management practices; nutrient management; and differences in other factors. This is similar to differential sensitivities of crop yields to changes in temperature and precipitation demonstrated in the literature^6,7^. If this is the case, the nationally pooled curves would moderate these opposing effects. One way to approach this is conducting a similar exercise on a single county, which allows our analyses to be fixed in space, and vary temporally. To further investigate this, a representative county was chosen for each crop which had maximum data records available and similar analyses were conducted. Supplementary Figure S2 presents crop yield residuals regressed against seasonal GDD for all crops, but for a single representative county for each crop species (maize: Antelope County, NE; soybean: Lawrence County, IN; sorghum: Montgomery County, KS; cotton-pima: Pinal County, AZ; cotton-upland: Tulare County, CA; winter wheat: Laramie County, WY; spring wheat-durum: Spink County, SD; and spring wheat-non-durum: Flathead County, MT). One striking difference that arises in this analysis is the increased R^2^ values, which range from 0.06 to 0.33. This signifies that crop yields for individual counties have a more pronounced response to GDD than what is interpreted from national-level curves. The natures of the relationships remained the same for both county and national scales (positive for all crops, except cotton).

**Figure 9:**
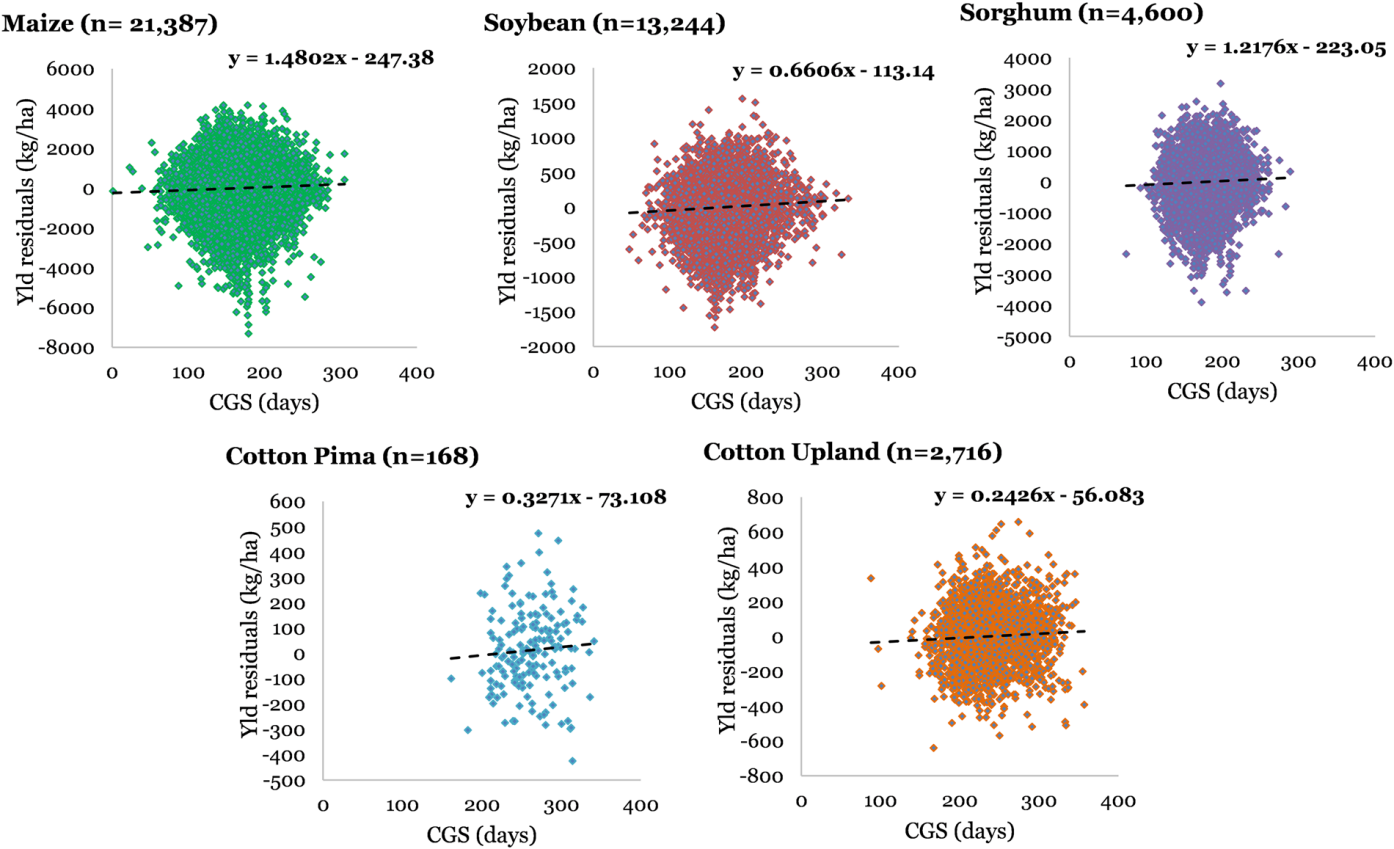
County-level relationships among yield and climatological growing season (CGS) pooled nationally for maize, soybean, sorghum, and cotton (pima and upland). Each regression curve includes *n* number of site-years in the CONUS during 1900–2014.

In a similar manner as mentioned above, we also explored relationships among county-level crop yield residuals and climatological growing season (CGS) length for the available site-years. Figure 9 presents these relationships, which are pooled for the CONUS under various crops. Spring wheat and winter wheat were excluded from this exercise, because their growing seasons generally include the frost/dormant periods, which are different than maize, soybean, and sorghum, hence it would not be worthwhile, at least for this study, to determine their relationships with frost-free period/CGS. For all crops, the crop yields had a positive response to increasing length of CGS. The R^2^ values, as with GDD, were low (<0.01) and hence not presented. This was, again, attributed to the spatial and temporal pooling of pairwise data, leading to aggregation of county-specific yield responses to CGS. When randomly-selected individual counties (maize: Pawnee County, NE; soybean: Vermillion County, IL; sorghum: Custer County, OK; cotton-pima: Pima County, AZ; and cotton-upland: Kern County, CA) were investigated for these relationships (Supplementary Figure S3), a similar relationship was observed as with GDD. The relationships yielded higher R^2^ values (up to 0.24) and we even found that maize yields showed negative response to increasing CGS, which is contrasting to the inference from the nationally pooled maize yield-CGS relationship, which had positive response. A very limited number of studies have looked into relationships of crop yield vs. agroclimate (GDD and CGS), and one study^4^ has reported correlation coefficients of −0.013 and 0.318 for Nebraska maize yield vs. growing season length and Nebraska maize yield vs. GDD, respectively. Their statistics (from Nebraska data) are somewhat comparable to our estimates from representative county analysis, because of similarity of scales, although they used state-level data as opposed to our county-specific approach. Moreover, they did not attempt to conduct their analysis on national scale, which is a knowledge gap our study has fulfilled. This finding further lends strength to our argument that the response of individual counties to changes in agroclimatic variables can vary spatially, both in nature and magnitude of sensitivity. Thus, while generalized assessments from national, continental, and global scale data can provide important inference for various applications to broader-level policy and decision-making, they would not be an accurate representation of individual county or finer scale trends and magnitudes in agroclimate vs. crop yield relationships for local policy and decision-making or strategy development.

Relatively weaker correlation between crop yields vs. CGS than crop yields vs. GDD, as inferenced from Figures S2 and S3 can be due to several reasons. Firstly, in practice, the spatial variation in CGS has led producers to adopt crop hybrids which suit a given site’s environment. For example, relative maturity of a maize hybrid planted in North Dakota may be around 80 days whereas for a hybrid planted in Texas, it may be up to 125 days. This explains why there is no observed north-south yield trend, similar to CGS (Fig. 5). This, however, doesn’t necessarily mean that the producers have also adopted to temporal changes in the CGS. Although it has been demonstrated that maize planting dates have shifted by approximately 2 weeks earlier relative to the early 1980’s^8^, this is not true for all crops and all regions considered in this study. Furthermore, even in a given state, producers may or may not plant different maturity groups of crops as a function of climatic gradients. Hence, crop yields might be slightly affected by variability in CGS in regions which have not adopted suitable varieties, as shown in Fig. 9. This low magnitude of crop response to CGS is expected because crop growing season tends to be narrower than the actual CGS in that region, and hence doesn’t affect crop yields. Even when crop failure occurs due to an early or later frost, producers usually opt for replanting, and hence may still get reasonable yields. GDD, shows a stronger response in crop yields, as it represents the actual daily growing conditions of the crop, while CGS merely represents a window around the crop growing season and hence, is responsible for affecting crop yields only in extreme scenarios (early or delayed frost).

## Discussion

The maps, data, and information, pertaining to the geographic and temporal variability associated with GDD, frost dates, and CGS are a resource that can act as one of the longest-term (115-year) climatology and trend information for these indices in the CONUS. The spatial patterns presented can aid users to study agroclimatic variability and their magnitudes as well as their potential implications to agriculture across regions of interest. Overall, the spatial patterns of agroclimatic indicators were found to be complex and region-specific. Trends in GDD, for example, can be thought of two contrasting natures in the CONUS, where GDD increased in the west (even higher trends in the southwest), and decreased in the Great Plains area and the east. This is in contrast to the spatial trends in GDD, which follow a temperature-induced north-to-south trend. Trends in monthly GDD are also somewhat similar, with regions of increasing and decreasing trends varying to some extent. Trends in annual frost dates, however, were found to be relatively more consistent nationwide, where FFF was observed to occur later and LSF occurred earlier. Some exceptions existed in the south and central southwest U.S., which showed trends that were in contrast to the rest of the CONUS. Due to predominant positive and negative trends in FFF and LSF respectively, CGS was observed to lengthen throughout the CONUS, with the exception of the same regions mentioned above. Thus, in this study, CONUS agroclimate was characterized as one where a lengthened climatological growing season prevails, and the heat accumulation within the crop growing season has increased and decreased in western and eastern U.S., respectively. These changes would affect crop management decisions such as planting dates and crop variety choices as well as expansion of crop planted area into regions with shorter CGS otherwise. Other comparative studies^3–5,9–11^, which focus on agroclimatic changes in the U.S., report similar results, although some of them vary in their choice of study periods, approach, indicators used etc., and also most of them did not explicitly focus on changes in climatic indices on agricultural production and practices.

This changed agroclimate can substantially impact agricultural decisions, operations and crop performance. It would also have substantial impact(s) on crop water use and irrigation requirements; nitrogen fertilizer use; pesticide, insecticide, fungicide use; changes in planting population density; and other management practices, A lengthened CGS could shift the agricultural belts northward, with greater opportunity to grow crops with lesser incidences of frosts. Moreover, areas with longer CGS (the southern regions), given the further lengthening, could be made suitable for double cropping and increase in harvesting frequencies^12^. With the earlier occurrences of LSF in the CONUS, earlier planting dates could be adopted, which has already been demonstrated for U.S. maize production^8^. It has also been reported^13^ that these earlier planting trends have led to 19–53% of state-level yield increases in U.S. maize production, and each additional day of earlier planting contributes to yield increases of 0.06–0.14 Mg ha^−1^. In this regard, a longer growing season also demands for a greater focus towards a greater need for water availability. Hence, a detailed spatial and temporal analysis of water deficits (availability) should be carried out within the framework of this study to have a better understanding of historical water-availability vs. growing season length dynamics and their impacts on crop water requirements. Relevant studies show that in the U.S. Great Plains in general, total precipitation amounts have been increasing and evaporative demand has been decreasing during 1968–2013^14^, although there is spatial non-uniformity in trends. Thus, there is likelihood that the longer CGS can be sustained with increased moisture availability, at least in the U.S. Great Plains. Planting longer season hybrids, varieties, or cultivars usually results in greater yields than medium and short season ones. Thus, when the resources and targeted yields are properly planned and managed, a lengthened growing season has the potential to be more economically beneficial to individual producer and to the national economy, in general. Moreover, a longer CGS would most likely be particularly beneficial (higher biomass accumulation) for perennial crops such as grass pastures, switchgrass, hay etc., because CGS in their case, is the actual growth window, unlike cereal crops, which have narrower-than-CGS growing seasons. In contrast, a longer growing season can be favorable to increased insect/pest and disease pressure^15–17^. For regions with a shortened growing season, however, there is a possibility that planted crops may not completely mature, resulting in lower yields and economic hardship. But, this does not seem to be the case for any of the crops at the national scale (Fig. 9). Through our county-specific results presented in Figure S3, we find that maize in Pawnee County, NE, does show a negative response to CGS lengthening, but this may not be the case for all maize growing counties, and calls for a detailed county-specific analyses for all the relevant counties in the CONUS.

GDD-crop yield interactions were analyzed in a similar manner, and it was found that on a national scale, crop yields were negatively associated with GDD accumulated during their growing seasons, except for cotton. Also, we observed that for crop-specific growing seasons (listed in Table S1), AGDD decreased with time for all crops, except spring wheat. These two observations lead to an inference that, in general, maize, soybean, sorghum, and winter wheat yields benefitted during the century, given that these show negative response to increasing GDD (Fig. 8). On the other hand, spring wheat and cotton yields were negatively impacted which for spring wheat, is attributable to observed increasing trends in GDD and the negative response of yields to GDD increases, while for cotton, is due to observed negative GDD trend, and the positive yield-GDD increase relationship. Thus, this study is important in another sense that is identifies and, perhaps more importantly quantifies these dynamics. We also calculated the change in crop yield caused due to changes in GDD and CGS per century for the chosen representative counties for each crop and U.S. pooled data (Supplementary Table S3). This was quantified using the slopes of the crop yield vs. agroclimate relationships and the observed agroclimate trend (Figs 8, 9, S2 and S3). It was observed that the changes calculated at the representative counties were greater than the ones obtained at U.S. pooled scale. Various cropping regions and cropping systems experience variable GDD trends, and moreover, show different responses of crop yields to these GDD trends, hence making the actual impacts site and crop specific. Furthermore, as we demonstrated earlier that crop yield vs. GDD relationships are better investigated at finer scales, we may also see variable responses within a given crop belt. Thus, there is a fundamental need for studying and optimizing our cropping regions in a way that we gradually shift towards areas which are more robust, resilient, and sustainable to these agroclimatic changes as well as experience beneficial agroclimate trends.

The trends in the CONUS agroclimate with respect to agricultural production, in conclusion, can be characterized by decreased heat accumulation during a fixed crop growing season for the majority of commodity crops, and lengthening of the climatological growing season for all crops studied. This implies that these two agroclimate indicators, counter each other as a lengthened CGS means increased availability of heat accumulation (in cases where producers and managers actually adapt to a longer CGS), whereas heat accumulation over time has decreased, which results in longer time (seasons) required for crop maturity. Hence, the actual crop yield impacts that different cropping regions have experienced would be dictated by a complicated balance between the lengthening of CGS and the decrease in heat accumulation. As a further study, the economics of these agroclimate-caused crop yield impacts should also be taken into consideration in the assessment of agroclimatic implications. We identified and suggest some valuable additions to the future work following our current study. Firstly, as observed, our analyses of crop yields vs. agroclimate suggest that it is better to investigate these relationships on finer (county) scales, rather than pooling data nationally. This was suggested based on the observance of loss of information caused by data aggregation. Our future work would include quantitatively estimating crop-yield vs. agroclimate relationships for individual counties independently, which we’ve demonstrated for one county for each crop species (Figures S2 and S3) in the present study. Secondly, the subject of this paper is limited to temperature-related agroclimatic indictors, and the questions at hand demand that these should be studied along with precipitation (or moisture), water deficit (precipitation relative to evaporative demand) and radiation-based indicators too, as these are also important driving variables for crop production in addition to temperature. Hence, although we showed that the changes in climatological growing season length and heat accumulation had benefits for the studied cropping systems, we suggest and plan to include other important agroclimatic indices in our future efforts. These can include diurnal temperature range, insolation, reference evapotranspiration, aridity index and water deficit. We acknowledge that these are important potential additions but need to be addressed in a comprehensive manner in a separate study similar to this. Although there are significant additions that could be implemented in this study, we maintain that the included efforts (maps, information, data, and interpretation) presented in this study are valuable owing to their period of study (115 years) as well as addressal of crop-specific agroclimate trends and impacts. These comprehensive analyses are crucial for studying local changes of agroclimate and its relation to cropland productivity for decision making by the climate and agricultural policymakers in the U.S. and the current study makes contribution to the scientific literature as well as for the policy and decision makers in practice.

## Material and Methods

### Data

The climate datasets for 1218 United States Historical Climatology Network sites (shown in Figure S4) for their entire data collection periods were retrieved from the National Centers for Environmental Information-National Oceanic and Atmospheric Administration (NCEI-NOAA). The dataset includes daily maximum and minimum air temperatures and daily precipitation. Since the study aims to analyze long-term trends in various agroclimatic indices, we limit our analyses to those weather station sites which have collected data for almost the whole study period (i.e., 1900–2014). For any missing daily information detected in a month, we did not calculate any index for that particular month, to avoid any misinformation generated from missing data estimation procedures in our analyses.

### Computation of agroclimatic indices

The study employs fundamental agroclimatic indicators, calculated at a daily time scale, for the entire study period, to detect trends that have occurred during that period. All of the observed variables and derived indices were converted to monthly (and annual) values by averaging or aggregating them as appropriate. This section describes the computation of the indices investigated in our study.

Each cropping system (maize, soybean, sorghum, cotton, and spring and winter wheat) has different growing season. Thus, the values growing degree days (GDD), or thermal units, were computed for each crop separately as the accumulation of T_avg_ exceeding a base temperature for each crop as:

1 $$GDD=(\frac{{T}_{{\max }}+{T}_{{\min }}}{2})-{T}_{base}$$

The base temperature used for various crops and their sources in the literature^18–29^ are listed in Supplementary Table S1. Two annual dates were identified for analysis: last spring frost (LSF) and first fall frost (FFF). Annual LSF date was defined as the latest day of the year before 15 July when T_min_ ≤ 0 °C. Similarly, annual FFF date was the earliest date after 15 July when T_min_ ≤ 0 °C. We also discuss the trends in the length of the climatological growing season—the period from LSF to FFF.

### Development of spatial agroclimate data

The agroclimatic indices calculated at the 1218 sites were represented in a spatial manner using geographic interpolation techniques. In this study, we used inverse distance weighing (IDW) interpolation technique, which was implemented using the Spatial Analyst Toolset in ArcGIS 10.4.1. IDW is a deterministic interpolation technique, where weights are assigned to point estimates using a mathematical function. The principle behind the IDW technique is that point estimates lying in closer vicinity of the prediction location will be more influential than the ones farther away. The algorithm followed by the IDW interpolation technique to determine the value of the variable of interest at unknown location (*Z* (*S*_*o*_)) is:

2 $$Z({S}_{o})=\sum _{i=1}^{N}{{\lambda }}_{i}Z({S}_{i})$$

where *S*_*o*_ is the location at which the value is to be predicted and *Z*(*S*_*o*_) is the value for the prediction location *S*_*o*_, *S*_*i*_ is the *i*^th^ location and *Z* (*S*_*i*_) is the known value at the *i*t^h^ location, *λ*_*i*_ is an unknown weight for the known value at the *i*^th^ location (equation 3).

3 $${\lambda }_{i}=\,\frac{{d}_{i}^{-p}}{{\sum }_{i=1}^{N}{d}_{j}^{-p}}$$

where *N* is the total number of known points to be used for the interpolation technique, *d* is the distance of the unknown value location from the known value location, and *p* is a power parameter. The significance of the power parameter (*p*) is that its magnitude governs the assignments of weights to the points. A higher *p* value results in more weight being assigned to closer points, which means a less smooth gridded surface. On the other hand, a lower *p* value assigns relatively lower weights to closer points, which results into a much smoother surface. For the purpose of this study, the value of *p* was optimized using ArcGIS 10.4.1.

This methodology was employed to develop gridded surfaces for all the agroclimatic indices (GDD, LSF, FFF, length of the climatological growing season). The point-based magnitudes of these indices over the 115-year study period were used as an input to the IDW interpolation tool. For analysis and inter-comparison on national scale, climate regions, and agricultural belts, it was essential to upscale site-specific indices. To extract these zonal values, zonal statistics tool in ArcGIS 10.4.1 was used. Hence, we were able to upscale site-specific indices to represent national and regional magnitudes.

### Trend detection

The Mann-Kendall (M-K) test, also referred to as Kendall’s tau test, is one of the most widely accepted non-parametric tests to detect significant trends in a time-series^30,31^. The null hypothesis (*H*_*o*_) stated by the M-K test is that a sample of data *X* = {*x*_*i*: *i*_ = 1, 2, … *n*}, *x*_*i*_ is a sample of *n* independent and identically distributed random variables. On the other hand, the alternative hypothesis *H*_*1*_ is that a monotonic trend exists in *X*. The test statistic *S* is asymptotically normal, has a mean zero and a variance which is computed using following equations;

4 $$S=\,\sum _{k=1}^{n-1}\sum _{j=k+1}^{n}sgn({x}_{i}-{x}_{j})$$

where, *x*_*j*_ are the sequential data values, *n* is the length of the dataset (number of data points).

5 $$\begin{array}{c}\,{sgn}({x}_{j}-{x}_{k})=\{\begin{array}{l}+1\,if\,({x}_{j}-{x}_{k}) > 0\\ \,\,0\,if\,({x}_{j}-{x}_{k})=0\end{array}\\ \,\,\,\,\,\,\,\,\,-1\,if\,({x}_{j}-{x}_{k}) < 0\end{array}$$

6 $$Var(S)=\,\frac{[n(n-1)(2n+5)-\,{\sum }_{t}\,t(t-1)(2t+5)]}{18}$$

where, *t* represents the extent of a given tie, and *Σ*_*t*_ is the summation over all ties. In cases where the sample size *n* > 10, the standard normal variable *Z* is computed using Eq. (7).

7 $$Z=\{\begin{array}{ll}\frac{S-1\,}{\sqrt{Var(S)}} & {\rm{if}}\,{\rm{S}} > 0\\ 0 & {\rm{if}}\,{\rm{S}}=0\\ \frac{S+1\,}{\sqrt{Var(S)}} & {\rm{if}}\,{\rm{S}} < 0\end{array}$$

Increasing trends are represented by positive values of *Z*, while decreasing trends are represented by negative values. In order to investigate the increasing or decreasing monotonic trends at the *α* significance level, the null hypothesis was rejected when the absolute value of *Z* greater than *Z*_*1-α/2*_ was detected, where *Z*_*1-α/2*_ was obtained from the standard normal cumulative distribution tables. The detection of any increasing or decreasing trends in this study was performed at the significance level of *α* = 0.05.

After the establishment of the fact that a linear trend is present in a particular time-series, a simple non-parametric procedure is applied to calculate the true magnitude of the slope of the linear trend^32^. This estimate is given by the Theil-Sen Estimator as:

8 $$b=Median\,(\frac{{x}_{j}-\,{x}_{z}}{j-1})$$

Considering an annual time series, *b* denotes the annual increment under the hypothesis of a linear trend. *b* provides the real slope of the tendency, and can vary slightly from the slope obtained from linear regression.

### Conus agricultural belts and climate regions

The major crop belts that were used to report trends in agroclimatic variables were obtained from National Centers for Environmental Information-National Oceanic and Atmospheric Administration (NCEI-NOAA). These belts were demarcated based on U.S. climate divisions, which were the building blocks of these belts. NCEI-NOAA was used as a source for maize, soybean, winter wheat, spring wheat, and cotton belts, whereas sorghum growing region was adopted from the USDA. These belts have been shown in the Supplementary Figure S5. The second monitoring reference we used in this study are the U.S. Climate Regions, which basically are nine climatically consistent regions in the CONUS. These have also been obtained from NCEI-NOAA and are shown in the Supplementary Figure S6.

### Determination of crop yields vs. agroclimate relationships

For developing relationships among crop yields and agroclimatic variables (GDD and CGS), residuals for crop yields were computed against time for the period from the start of data records until 2014. Secondly, we identified counties where at least one USHCN weather station site physically existed and we used that particular station(s) (averaged where multiple sites were identified) to better represent agroclimatic conditions in that county. Next, we narrowed down counties used for analyses to the ones that had at least 75% of crop yield data records (residuals) or a maximum of 25% missing data records.

GDD accumulated during a particular crop’s growing season were determined by adding daily GDD for that period at each USHCN site. The usual crop planting and harvesting dates (or months) were adopted from NASS-USDA Agricultural Handbook Number 628 published in October 2010. The planting and harvesting dates considered for each crop’s growing season are listed in the Supplementary Table S1. These resultant crop total GDD magnitudes were used to develop crop yield vs. GDD relationships. For crop yield vs. CGS relationships, we used the computed CGS for each year and each USHCN site.

Finally, crop yield residuals and agroclimate indicators (either GDD or CGS) were used in pairwise comparison to conduct a linear regression analysis for all available counties for a particular crop to develop a nationally pooled relationship. Similar procedure was performed to demonstrate/analyze county-specific relationships.

## Electronic supplementary material

supplementary information
