# Supplementary material for: U.S. Agro-Climate in 20th Century: Growing Degree Days, First and Last Frost, Growing Season Length, and Impacts on Crop Yields
Source: Sci Rep. 2018 May 3;8:6977. doi: 10.1038/s41598-018-25212-2 (PMC5934404; doi:10.1038/s41598-018-25212-2)
Supplement: Supplementary file 1 — supplementary information [file 41598_2018_25212_MOESM1_ESM.pdf]

# U.S. AGRO-CLIMATE IN 20$^{TH}$ CENTURY: GROWING DEGREE DAYS, FIRST AND LAST FROST, GROWING SEASON LENGTH, AND IMPACTS ON CROP YIELDS

**Meetpal S. Kukal[1] and Suat Irmak[1]***

University of Nebraska-Lincoln, Lincoln, NE 68583, U.S.A. *Corresponding author's e-mail: sirmak2@unl.edu. Address: 239 L.W. Chase Hal, Lincoln, NE, U.S.A., 68583.

## SUPPLEMENTARY INFORMATION

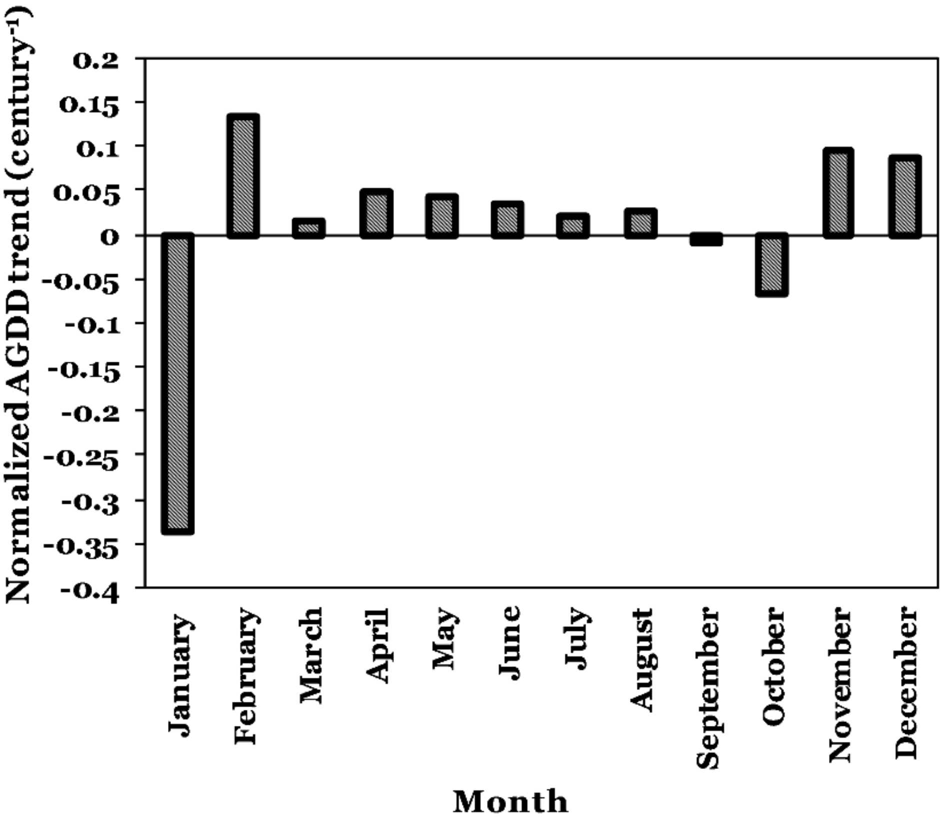


**Supplementary Figure S1.** Normalized monthly AGDD temporal trends (century$^{-1}$) averaged for the entire CONUS.

**Supplementary Table S1. Planting and harvesting dates and base temperature ($T_{base}$) considered for growing degree days for each crop studied.**

| Crop | Planting | Harvesting | Base temp. (℃) | Sources that aided selection of base temperature |
|---|---|---|---|---|
| **Maize** | 1-Apr | 30-Nov | 10 | Djaman and Irmak (2013); Irmak et al. (2012) |
| **Soybean** | 1-May | 30-Nov | 10 | Payero et al. (2005); Irmak et al. (2013) |
| **Sorghum** | 1-May | 30-Nov | 10 | Anda and Pinter (1994); Craufurd et al. (1998) |
| **Cotton** | 1-Apr | 30-Nov | 10 | Sammis et al. (1985); Bange and Milroy (2004) |
| **Winter wheat** | 1-Sep | 31-Jul | 0 | McMaster and Wilhelm (1997); Baker and Gallagher (1983) |
| **Spring wheat** | 1-Apr | 30-Sep | 10 | Petr J (1991); Slafer and Savin (1991) |

## Supplementary Table S2. Long-term mean agroclimate indicators for CONUS and U.S. Climate Regions

| Variable | Units | | U.S. Climate Regions | | | | | | | | |
|---|---|---|---|---|---|---|---|---|---|---|---|
| | | CONUS | West | Upper Midwest | Southwest | Southeast | South | Ohio Valley | Northwest | N. Rockies and Plains | Northeast |
| **CGS** | Days | 167 | 182 | 139 | 155 | 221 | 220 | 177 | 134 | 123 | 147 |
| **FFF** | | 285 | 296 | 272 | 282 | 310 | 308 | 289 | 270 | 263 | 278 |
| **LSF** | | 116 | 108 | 132 | 125 | 83 | 86 | 111 | 136 | 139 | 129 |
| **Annual AGDD** | Degree C | 2026 | 2248 | 1380 | 1956 | 3132 | 3129 | 2122 | 1126 | 1271 | 1380 |
| **January AGDD** | | 15 | 18 | 0 | 6 | 60 | 36 | 5 | 1 | 0 | 1 |
| **February AGDD** | | 20 | 33 | 0 | 13 | 66 | 51 | 7 | 1 | 0 | 1 |
| **March AGDD** | | 50 | 68 | 5 | 37 | 133 | 122 | 37 | 5 | 4 | 7 |
| **April AGDD** | | 106 | 121 | 34 | 93 | 223 | 223 | 109 | 29 | 30 | 36 |
| **May AGDD** | | 213 | 222 | 136 | 200 | 353 | 357 | 239 | 99 | 113 | 135 |
| **June AGDD** | | 331 | 331 | 267 | 332 | 450 | 476 | 371 | 187 | 231 | 262 |
| **July AGDD** | | 428 | 455 | 359 | 433 | 510 | 551 | 450 | 310 | 355 | 351 |
| **August AGDD** | | 403 | 428 | 323 | 399 | 497 | 541 | 424 | 290 | 324 | 321 |
| **September AGDD** | | 273 | 311 | 182 | 272 | 405 | 414 | 299 | 161 | 163 | 195 |
| **October AGDD** | | 134 | 176 | 59 | 124 | 256 | 254 | 137 | 45 | 45 | 66 |
| **November AGDD** | | 43 | 59 | 5 | 29 | 123 | 100 | 33 | 4 | 2 | 12 |
| **December AGDD** | | 17 | 19 | 0 | 7 | 67 | 42 | 7 | 1 | 0 | 2 |

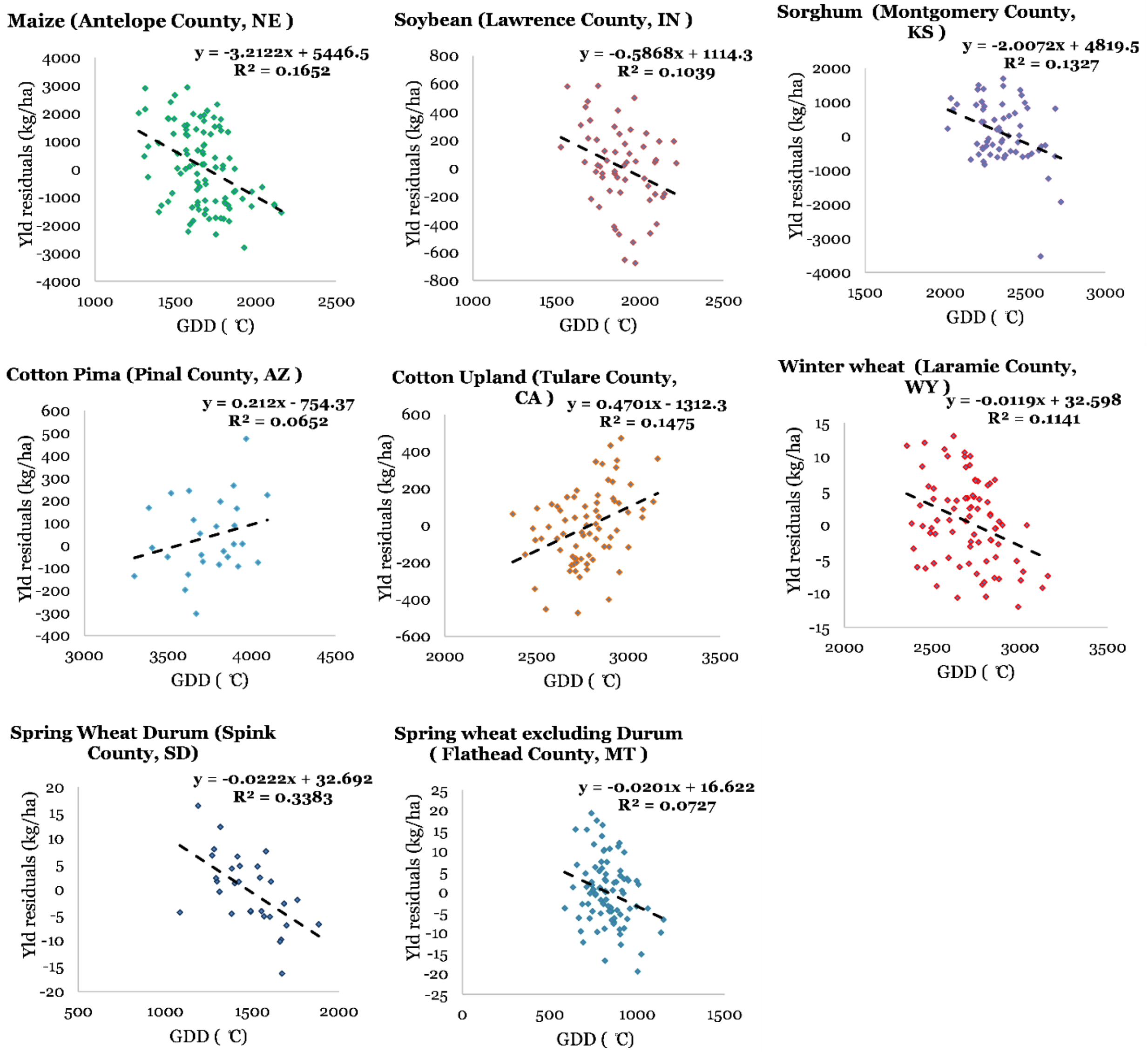


**Supplementary Figure S2.** County-level relationships among yield and growing degree days for one representative county for maize, soybean, sorghum, cotton (pima and upland), winter wheat and spring wheat (durum and non-durum). Each regression curve includes n number of site-years in the U.S. during 1900-2014.

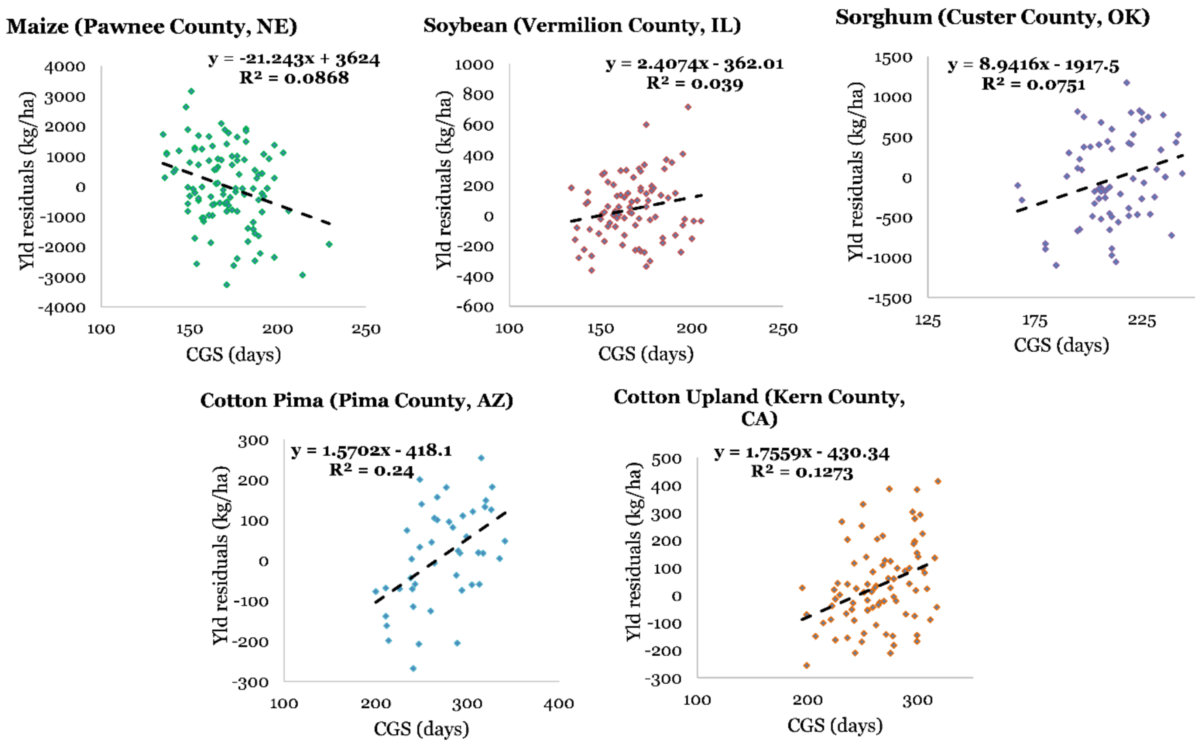


**Supplementary Figure S3.** County-level relationships among yield and climatological growing season for one representative county for maize, soybean, sorghum and cotton (pima and upland). Each regression curve includes n number of site-years in the U.S. during 1900-2014.

**Supplementary table S3. Change in crop yield caused due to changes in growing degree days and climatological growing season for counties pooled at the U.S. level and the chosen representative county.**

| Crop | Change in crop yield (kg ha$^{-1}$ century$^{-1}$) due to change in: | | | |
|---|---|---|---|---|
| | Growing Degree Days | | Climatological Growing Season | |
| | U.S. pooled | Representative county | U.S. pooled | Representative county |
| **Maize** | 0.2 | 301.3 | 6.7 | -404.2 |
| **Soybean** | 2.6 | 278.1 | 6.8 | 67.0 |
| **Sorghum** | 29.6 | 565.1 | 10.8 | 120.6 |
| **Cotton Pima** | -1.5 | -302.2 | 6.0 | 163.1 |
| **Cotton Upland** | -1.4 | 304.9 | 4.4 | 85.1 |
| **Winter wheat** | 2.1 | -3.2 | * | * |
| **Spring Wheat (durum)** | -72.3 | 6.0 | * | * |
| **Spring wheat (non-durum)** | -15.8 | 0.9 | * | * |

* represents that the impacts of climatological growing season were not investigated on wheat.

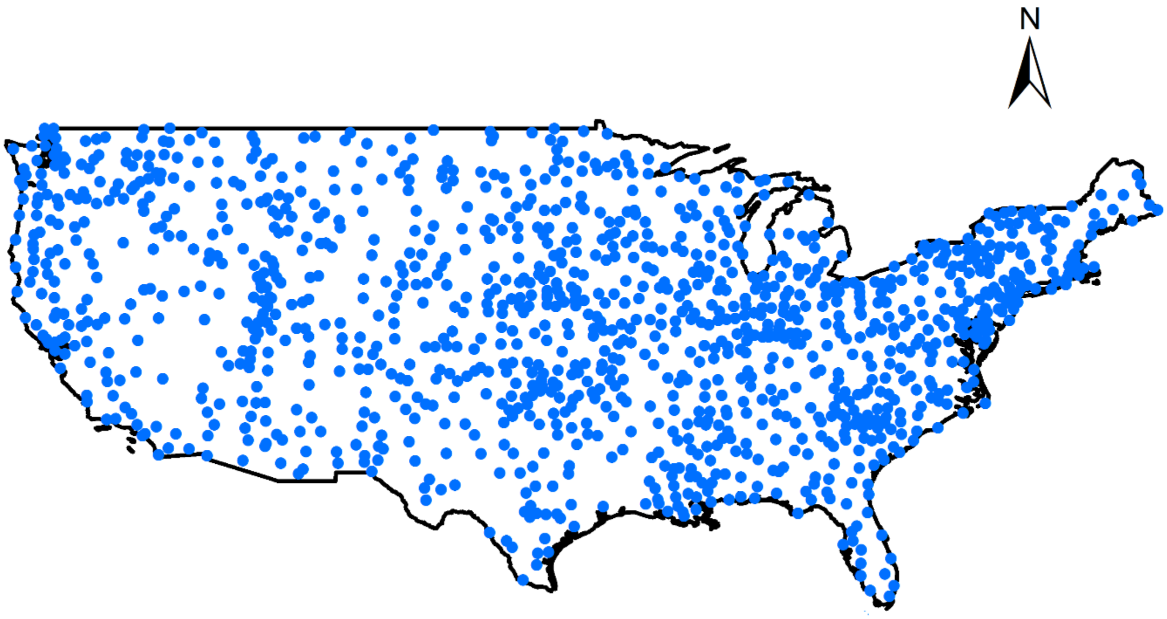


**Supplementary Figure S4.** United States Historical Climatology Network sites used to develop maps for various agroclimatic indices. The total number of sites are 1218. We created the map using ESRI ArcMap 10.4.1 software http://desktop.arcgis.com/en/arcmap/.

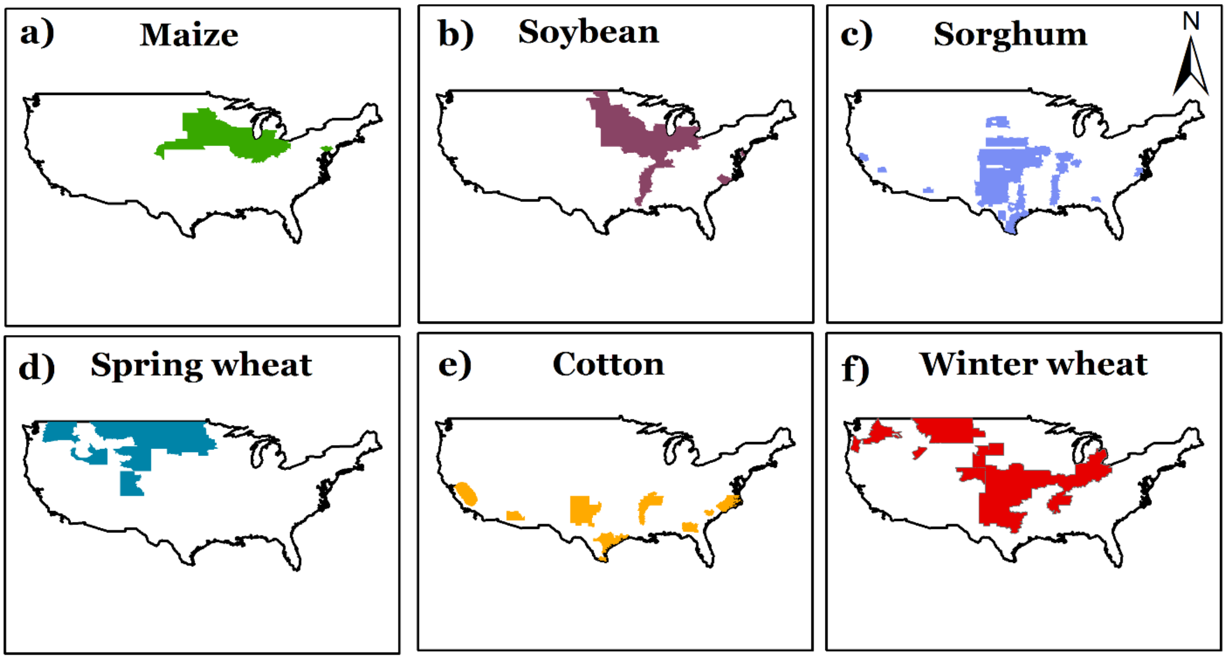


**Supplementary Figure S5.** The spatial distribution of major agricultural belts in the U.S. represented by colored regions in the U.S. map. All the agricultural belts were adopted from NOAA-NCEI, except sorghum, which was constructed from USDA. We created the maps using ESRI ArcMap 10.4.1 software http://desktop.arcgis.com/en/arcmap/.

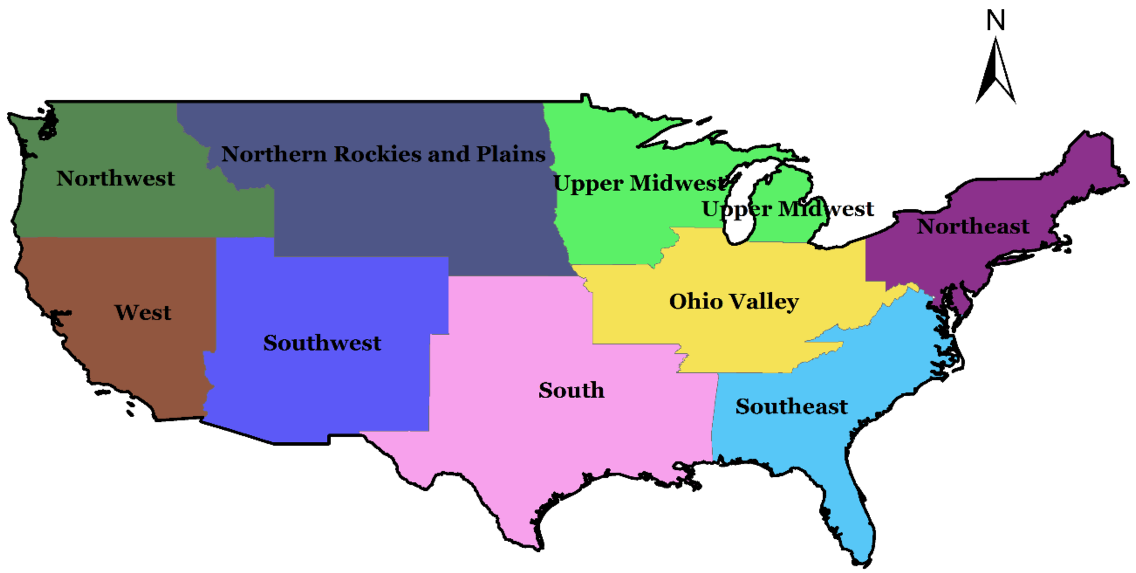


**Supplementary Figure S6.** U.S. climate regions defined by NCEI-NOAA. We created the map using ESRI ArcMap 10.4.1 software http://desktop.arcgis.com/en/arcmap/.
